# Dietary Wheat Amylase Trypsin Inhibitors Impact Alzheimer’s Disease Pathology in 5xFAD Model Mice

**DOI:** 10.3390/ijms21176288

**Published:** 2020-08-31

**Authors:** Malena dos Santos Guilherme, Victor F. Zevallos, Aline Pesi, Nicolai M. Stoye, Vu Thu Thuy Nguyen, Konstantin Radyushkin, Andreas Schwiertz, Ulrich Schmitt, Detlef Schuppan, Kristina Endres

**Affiliations:** 1 Department of Psychiatry and Psychotherapy, University Medical Center Johannes Gutenberg-University, 55131 Mainz, Germany; malena.dossantosguilherme@unimedizin-mainz.de (M.d.S.G.); nicolai.stoye@gmx.net (N.M.S.); vuthuthuy.nguyen@unimedizin-mainz.de (V.T.T.N.); 2 Institute of Translational Immunology and Research Center for Immune Therapy, University Medical Center, 55131 Mainz, Germany; victor.zevallos@northumbria.ac.uk (V.F.Z.); alinpesi@uni-mainz.de (A.P.); 3 Nutrition and Food Research Group, Department of Applied and Health Sciences, University of Northumbria, Newcastle Upon Tyne NE1 8ST, UK; 4 Leibniz Institute for Resilience Research, 55122 Mainz, Germany; Konstantin.Radyushkin@lir-mainz.de (K.R.); ulrich.schmitt@lir-mainz.de (U.S.); 5 Institute of Microecology, 35745 Herborn, Germany; Andreas.Schwiertz@mikrooek.de; 6 Division of Gastroenterology, Beth Israel Deaconess Medical Center, Harvard Medical School, Boston, MA 02115, USA

**Keywords:** Aβ, ATI, 5xFAD, gluten, inflammation, intestine, microbiota, plaque, TLR4, wheat sensitivity

## Abstract

Wheat amylase trypsin inhibitors (ATIs) represent a common dietary protein component of gluten-containing cereals (wheat, rye, and barley). They act as toll-like receptor 4 ligands, and are largely resistant to intestinal proteases, eliciting a mild inflammatory response within the intestine after oral ingestion. Importantly, nutritional ATIs exacerbated inflammatory bowel disease and features of fatty liver disease and the metabolic syndrome in mice. For Alzheimer’s disease (AD), both inflammation and altered insulin resistance are major contributing factors, impacting onset as well as progression of this devastating brain disorder in patients. In this study, we evaluated the impact of dietary ATIs on a well-known rodent model of AD (5xFAD). We assessed metabolic, behavioral, inflammatory, and microbial changes in mice consuming different dietary regimes with and without ATIs, consumed ad libitum for eight weeks. We demonstrate that ATIs, with or without a gluten matrix, had an impact on the metabolism and gut microbiota of 5xFAD mice, aggravating pathological hallmarks of AD. If these findings can be translated to patients, an ATI-depleted diet might offer an alternative therapeutic option for AD and warrants clinical intervention studies.

## 1. Introduction

Currently about 30 million people worldwide suffer from Alzheimer’s disease (AD) and for the vast majority, the sporadic cases, the origin of the disease stays still enigmatic. Experimental therapeutic drugs mostly showed high efficacy at least in rodent models of the disease (for an overview see, e.g., [1]); however, as its onset settles decades before clinical signs occur, pharmacological treatment of humans might not present the best strategy due to prolonged exposure and side effects. A better approach would be to give lifestyle advice that can be integrated into daily life and have in general already been shown to benefit cognition in people at risk of developing dementia (FINGER study, multidomain lifestyle intervention, [2]). However, underlying mechanisms are still not fully understood. Nutritional compounds such as linoleic acid, vitamins or polyunsaturated fatty acids have been discussed as being preventive for decades, accordingly (for example: [3]). Many of these nutritional substances are correlated to immune modulatory probabilities and AD patients also have been demonstrated to show altered immune stimulation [4,5] as well as mutations in microglial genes such as triggering receptor expressed on myeloid cells 2 (TREM2; [6]). Therefore, assuming a causative role for immune modulatory nutritional substances would be a reasonable hypothesis (for a recent review see [7]). An increasing body of evidence suggests that not only the inflammatory status in the brain might be relevant but also peripheral inflammation. In this regard, the gut might play an outstanding role with a large surface exposed to a plethora of microbial organisms and nutritional substances. It has been shown that changes in nutrition and/ or the intestinal microbial community can affect gut morphology, neuroinflammation, and even neurodegeneration (for a recent example see: [8]). Amylase trypsin inhibitors (ATI) are commonly ingested immune stimulatory proteins, mostly present in dietary wheat, rye and barley. They appear to be more relevant than the abundant gluten itself as triggers of the multifaceted nonceliac wheat sensitivity (NCWS) syndrome. NCWS is characterized by a plethora of intestinal and extraintestinal symptoms elicited by dietary wheat proteins (especially gluten and ATIs), after exclusion of inflammatory bowel disease, wheat allergy, and celiac disease [9,10]. ATIs represent 2–4% of wheat protein and have been found to not only inhibit trypsin and amylase [11] but also toll-like receptor 4 (TLR4) on monocytes, macrophages, and dendritic cells [12,13]. Thus, ATIs promoted intestinal inflammation in a rodent model of inflammatory bowel disease [13]. The role TLR4 plays in AD is not fully deciphered; a recent report found the minor allele of the rs4986790 polymorphism (G) of TLR4 to be associated with a reduced risk of developing AD and higher cortical thickness in human patients [14]. Low-dose ligands for TLR2 and TLR4 attenuated learning deficits in rats when administered before intracerebroventricular injection of Amyloid β (Aβ) peptides [15]. This was consistent with findings from Tau-transgenic AD model mice [16]. However, there is also one contradicting report that used a TLR4 antagonist and TLR4 knock-out mice that describes TLR4-dependent glial cell activation that led to impairment in memory establishment in mice [17].

To investigate the potential role of nutritional ATIs on AD progression, we fed male AD model mice (5xFAD) with dietary regimes containing different amounts of ATIs in a gluten-free or a gluten-containing matrix, namely: (1) Gluten- and ATI-free diet, (2) Low ATI (0.2% of chow) without gluten (3) Low ATI (0.04% of chow) with gluten (25% of protein) and (4) High ATI (0.6% of chow) with gluten (25% of protein). The four groups of animals were subsequently analyzed for intestinal morphometry, inflammatory markers, gut microbial composition, behavioral deficits, and plaque deposition.

## 2. Results

### 2.1. Food Intake and Body Weight Gain

**Figure 1:**
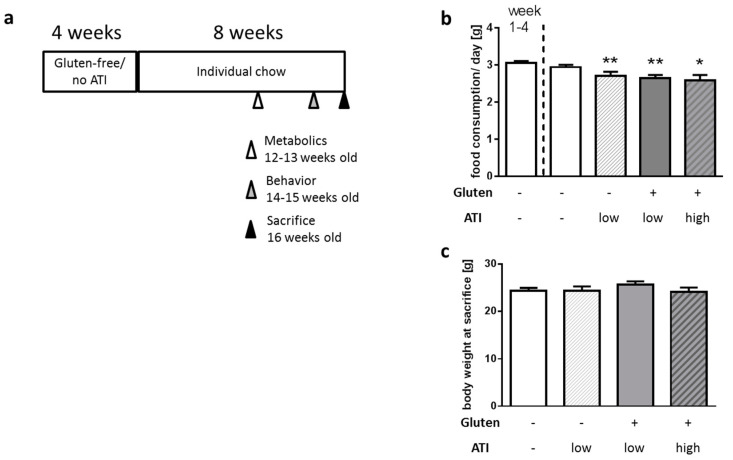
Acceptance of diet, and body weight development of 5xFAD mice fed with a standardized diet containing no, high or low amounts of wheat amylase trypsin inhibitors (ATIs) on a gluten-containing or gluten-free background. (**a**) Schematic representation of the diets and experimental regime. Mice were aged 4 weeks when all animals received the diet without gluten or ATI for 4 weeks to achieve a basal condition. Afterwards, animals were assigned to the four different diets for 8 weeks. Animals were sacrificed at a final age of 16 weeks. (**b**) Food consumption per day in the start phase (week 1–4) and on the different diets. (**c**) Body weight at sacrifice (*n* ≥ 8 per group; all data presented as mean + SEM; One Way ANOVA with Holm–Sidak’s multiple comparisons test; *, *p* < 0.05, **, *p* < 0.01).

5xFAD mice represent a genetic AD model carrying two mutations in human presenilin 1 and three in human APP [18]. Thereby, the model is relatively aggressive and plaques as well as behavioral deficits can already be detected early. A food preference test revealed that female and male mice react slightly different to the four types of diets used in our study (Figure S1): male mice clearly preferred gluten- and ATI-free food pellets, while females tended towards the gluten-containing diet, independently of its ATI content. Together with the prior finding that females of this strain show a faster disease progression (e.g., [19]), we here restricted the experiments to male mice to minimize potential measurement deviations. Animals were kept on a gluten- and ATI-free, well-defined diet (Table S1) for 4 weeks starting at the age of 4 weeks. Afterwards, they received the same nutrient-defined diet (1) without additions, or enriched with (2) 1% of the protein (casein) substituted by ATI, (3) 25% of the protein as gluten with a low ATI content (0.2%), or (4) 25% of the protein as gluten with a high ATI content (3%) for another 8 weeks (Figure 1a). Animals continuing on the original diet had a slightly higher food consumption than the other groups (Figure 1b) which can be explained by the preference of this food by male mice observed in the pre-test (Figure S1); however, this had no influence on the body weight of all four groups at sacrifice (age of 16 weeks; Figure 1c). This indicates that the different diets did not affect growth during puberty and young adulthood.

### 2.2. Physical Activity and Oxygen Consumption

**Figure 2:**
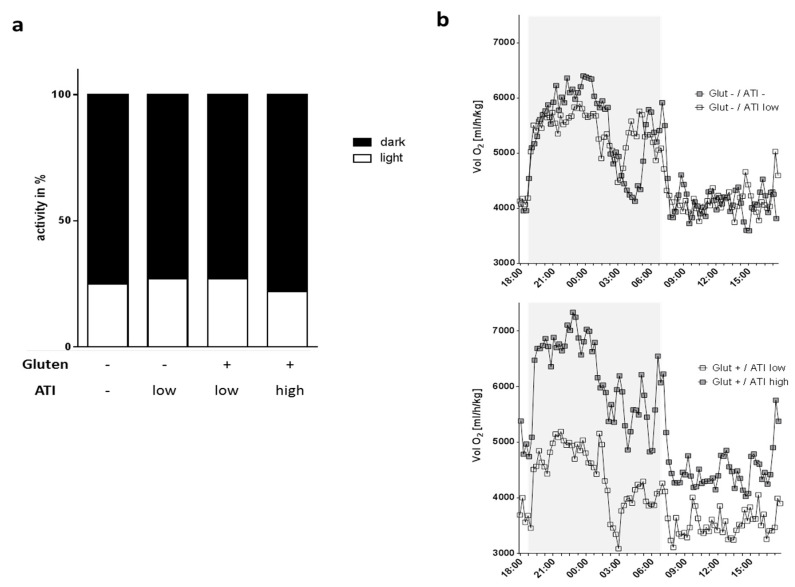
Activity and metabolic capacity of 5xFAD mice under the 4 different diets. (**a**) Locomotive activity during dark and light phase was assessed by light-beam sensors. (**b**) Oxygen consumption. Mice were placed in metabolic cages for 48 h and one cycle of measurement including 24 h was used for analysis (*n* ≥ 7 per group). The dark phase is indicated by a grey box. Shown are means per time point; SEMs and *p*-values of multiple t-test comparison are not shown for clearness of the graphs (*p*-values are given in Table S2).

AD is associated with disturbances of circadian rhythm [20,21] and with metabolic diseases such as type 2 diabetes [22]. In addition, tremendous shifts in energy demand and oxygen consumption have to be expected during AD-related inflammatory processes [23]. We therefore analyzed oxygen consumption as well as locomotion by using metabolic cages during a period of 24 h. Overall, day- and night-time activity patterns and general activity were not affected by the different diets in the 5xFAD mice (Figure 2a and data not shown). Mice showed an approximate relative activity of 73–78% in the dark phase which is in accordance with the expected negative masking of locomotor activity during the light period [24]. When comparing oxygen consumption, it became obvious that low dietary ATI on a gluten-containing background only slightly affected metabolic activity compared to mice on the gluten- and ATI-free diet (Figure 2b, upper graph, single *p*-values are reported in Table S2). On the contrary, gluten with the higher amount of ATI (3%) led to an increased need for oxygen when compared to mice fed with gluten and low ATI amount (Figure 2b, lower graph, single *p*-values are reported in Table S2). This suggests that the ATIs in the diet, supplemented at a concentration that is comparable to average human daily wheat consumption, induce significant increases in oxygen consumption.

### 2.3. ATI-Induced Changes in the Gut

**Figure 3:**
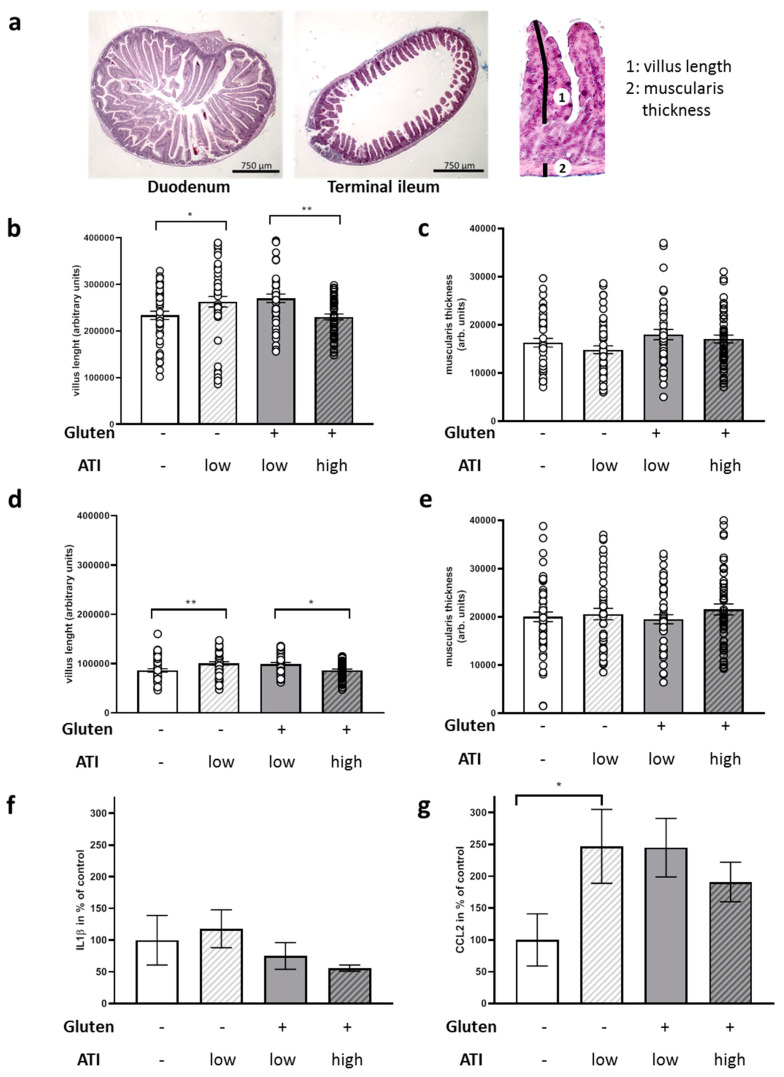
Micromorphological measurements and quantitation of inflammatory markers in the small intestine of 5xFAD mice fed with ATI-deficient or ATI-supplemented diet. Tissue specimens were drop-fixed and subsequently two slices per tissue sample and animal were used for HE staining. Length of two villi and the thickness of the underneath located muscularis were measured per slide. (**a**) Exemplary pictures of duodenum and terminal ileum. For an example of measures of villus length (1) or muscularis thickness (2), see the magnified segment of the terminal ileum on the right. (**b**) Relative measures of duodenal villi. (**c**) Thickness of muscularis from duodenum. (**d**) Villus length within terminal ileum sections. (**e**) Muscularis thickness within terminal ileum sections. All data are given as mean ± SEM. Statistical analysis was performed by One Way ANOVA (Sidak’s multiple comparisons test; *, *p* < 0.05; **, *p* < 0.01; *n* = 7 animals per group). (**f**) Interleukin 1 β (IL1β) and (**g**) C–C motif chemokine ligand 2 (CCL2) mRNA levels of terminal ileum were quantified by qPCR and normalized to beta actin mRNA levels. Data present mean ± SEM. Statistical analysis was performed by One Way ANOVA (Uncorrected Fisher’s LSD test; *, *p* < 0.05; *n* = 4–8 animals per group).

Muscularis thickness and villus length were assessed from the duodenum and terminal ileum as representative sections from the small intestine (Figure 3a). While the muscle layer was not affected with any of the diets (Figure 3c,e) in both tissue specimens, villus length increased when a low amount of ATIs was fed on a gluten-free background and mild villous atrophy occurred in animals fed on a gluten-containing diet with high amounts of ATIs as compared to low ATIs (Figure 3b,d).

Ileal transcript levels of the monocyte-macrophage activation marker interleukin 1 β (IL1β) were comparable in all mice, regardless of the dietary regime (Figure 3f), whereas C–C motif chemokine ligand 2 (CCL2), a chemokine recruiting monocytes and macrophages to sites of injury, was significantly increased in all ATI-fed groups, compared to the mice fed an ATI- and gluten-free diet (Figure 3g), in accord with our data in other models of disease [13,25,26].

**Figure 4:**
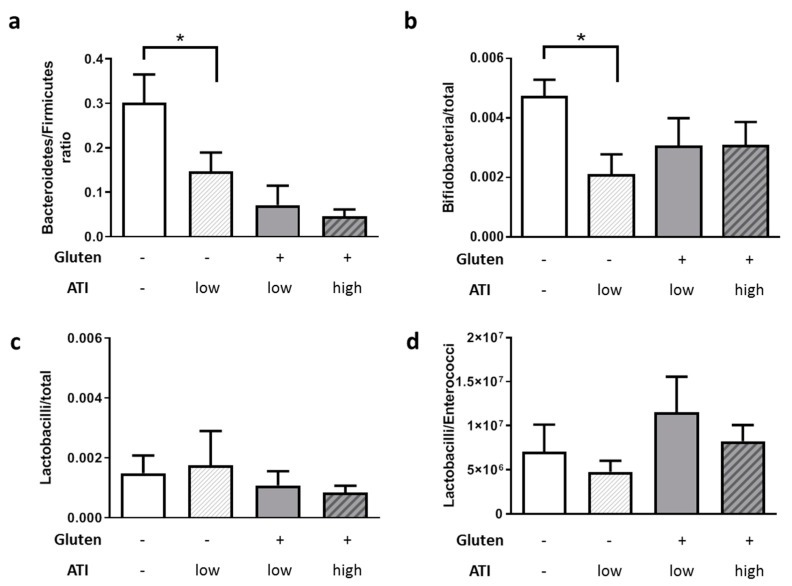
Fecal microbial communities in animals are changed by nutritional ATIs. Fecal samples were collected during sacrifice and subjected to quantitative PCR-based analysis. (**a**) Ratio of Bacteroidetes to Firmicutes. (**b**) Bifidobacteria in relation to total bacterial cell counts. (**c**) Lactobacilli in relation to total bacterial cell counts. (**d**) Ratio of Lactobacilli to Enterococci. All data are given as mean + SEM. Statistical analysis was performed by One Way ANOVA (Sidak’s multiple comparisons test; *, *p* < 0.05; *n* ≥ 6 per group).

Moreover, as shown in Figure 4a, ATIs led to a shift towards a lowered Bacteroidetes to Firmicutes ratio, with a clear dose-dependent effect and significant cumulative effect when combined with gluten (*p* = 0.0013 and 0.0003). Additionally, ATI reduced Bifidobacteria in line with our prior study in mice with inflammatory bowel disease [26] (Figure 4b). On a gluten-containing background, no further changes were measured and the Lactobacilli or the Lactobacilli/ Enterococci ratio appeared not to be changed at all by gluten (Figure 4c,d). The only difference between the mice fed with gluten with ATI high vs ATI low was a general reduction (*p* = 0.055) of total bacterial counts from 1.5 × 10^10^ to 5 × 10^9^/g (data not shown).

### 2.4. Impact of ATI on Hippocampus-Based Behavior

**Figure 5:**
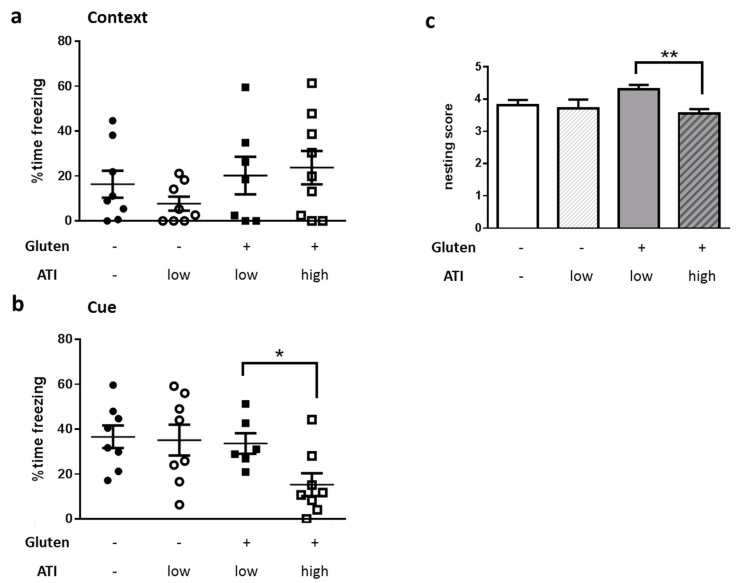
Influence of the quantity of ATI supplied in the diets on hippocampus-dependent tasks in 5xFAD mice. Mice were subjected to fear conditioning learning and memory recall in a context (**a**)- or cue (**b**)-dependent manner. (**c**) Nesting score. Mice were habituated to a special nesting material and nests were scored after overnight construction. All data are presented as mean with SEM. Statistical analysis was performed by pairwise Student’s t-test (*, *p* < 0.05; **, *p* < 0.01; *n* ≥ 6 per group).

After having demonstrated a direct effect of ATIs on intestinal properties, we next wanted to understand if feeding a diet with amounts of ATIs that are comparable to quantities consumed in human wheat-based populations also impacts performance in behavioral tasks that are indicative for AD. We chose fear conditioning and nest building as two tasks that are related to hippocampal function [27,28]. Context-dependent learning and memory was not distinguishable in all four groups of mice (Figure 5a). As we here also obtained several measurements of zero seconds freezing (freezing indicates the fear reaction), we have to acknowledge that context-dependent learning probably was not optimal with our chosen parameters. In addition, a diminished contextual learning has been described to only occur in older 5xFAD (starting with 6 to 7 months of age [29]). Nevertheless, the cue-dependent recall of fear, as measured by freezing time, was reduced from 35% to 15% in mean in animals fed on a gluten-containing diet with high ATI content as compared to those on gluten with low ATI (Figure 5b). Similarly, nest building ability was also reduced when comparing mice on a high ATI and gluten-containing with those on low ATI and gluten-containing diet (Figure 5c; material integrated into the nest was also lower in gluten+/ATI high versus gluten+/ATI low but did not reach statistical significance: *p* = 0.185, data not shown). The nesting score was reduced from 4.3 to 3.7 points, equivalent to a nest built as a cup with high walls or nearly an incomplete dome to a nest built with low to medium walls (for a picture of the scoring scale see [30]).

### 2.5. Influence of ATI on Pathological Hallmarks in the Brain

**Figure 6:**
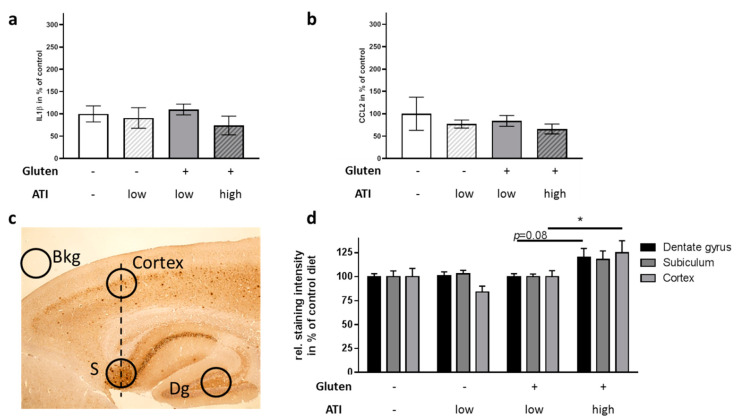
Inflammatory markers and plaque deposition in the brain of 5xFAD mice ingesting different levels of ATIs. (**a**) IL1β and (**b**) CCL2 mRNA levels were quantified by qPCR and normalized to beta actin mRNA levels. Data present means ± SEM. Statistical analysis was performed by One Way ANOVA (*n* = 4–6 animals per group). (**c**) Positioning of densitometric analysis areas on the brain slides (S: Subiculum; Dg: Dentate gyrus). Exemplarily, a slide derived from a mouse fed with gluten- and ATI-free diet is shown. (**d**) Quantitative analysis of Aβ-containing depositions. Measurements have been corrected for background (Bkg) and are presented as staining relative to area in % of gluten-free/ATI-free diet. Two slides per animal were analyzed (*n* = 5–8). All data are given as mean + SEM. Statistical analysis was performed by One Way ANOVA (*, *p* < 0.05).

Inflammation plays a major role in AD (for recent reviews see, e.g., [31,32]. As we already observed an increase in CCL2 in the small intestine, we next analyzed brain inflammatory status: neither CCL2 nor IL1β was significantly affected in whole brain lysates (Figure 6a,b). Cerebral Aβ accumulation in 5xFAD mice starts to increase already around the age of 2 months [18]. As this is the time-point when feeding on the respective diets started, and since the observed behavioral changes became significant after 6 to 7 weeks on the different diets, we assumed that the ATI-containing diet might have induced exacerbated histological hallmarks of AD at the time of sacrifice. Brain hemispheres were stained with the antibody 6E10, which selectively detects the heterologously expressed human APP products. Plaque density was assessed in cortical as well as in hippocampal areas (Figure 6c). On the gluten-free background, the low supplementation with ATIs revealed no influence on plaque deposition, neither in the cortex nor in the hippocampus (Figure 6d). However, when fed with the high ATI-containing diet together with gluten, plaque deposition was increased as compared to the other dietary groups. In the dentate gyrus and the subiculum this did not reach significance (*p* = 0.08 and 0.09), but mice on the gluten/high ATI diet displayed a significant increase of cortical Aβ-containing plaques to 125% as compared to all other groups on the ATI-free or the low ATI-containing diets.

## 3. Discussion

The crop component ATI, served with a normal gluten-containing diet, aggravated metabolic dysfunction, led to changes in intestinal architecture and to some extent decreased cognitive abilities of the 5xFAD model mice. This probably was based also on increased plaque deposition in distinct brain areas.

The underlying reason for the still increasing number of people with sporadic AD has not been identified. Various nutritional compounds have been discussed, such as lack of vitamins (e.g., [33]) or dietary lipids [34]. However, before most of the nutritional substances might exert their beneficial or detrimental effect, they have to pass the gastrointestinal system. Within recent years, for at least some of therapeutically discussed compounds for neurological or other diseases, the relevance of microbial metabolism to gain active molecules has been demonstrated [35,36]. Moreover, the first evidence has been found showing that peripheral immune reactions such as those within the gut, might affect also inflammatory or disease status of organs such as the brain (for example [37,38]; reviewed for AD in [39]). For AD, inflammation plays an important role even if the timeline of positive and negative immune reactions is not always fully clarified [40]. Therefore, we here tried to answer the question if ATIs could, via their pro-inflammatory properties, elicit AD-like deficits in a popular mouse model.

We restricted our investigations to male mice as we observed a preference for gluten-containing diet in female mice. In humans, food preference is culturally determined. It is well-known that women in western societies tend to have better knowledge with regard to food and nutrition (e.g., [41]) which consequently also leads to a higher intake of healthy food components (fruit and vegetables, dietary fiber and lower intakes of fat and salt [42]). In mice, a craving for food with high energy content would be expected but energy load was comparable in all four offered diets. No investigation about preference of food devoid of gluten or of gluten-containing food in mice could be identified from the literature. However, whey protein was substituted by casein in the gluten-free diet types. Hydrolyzed casein has been demonstrated to be avoided dose-dependently by mice [43] probably because of the bitter taste of some of the resulting peptides and amino acids. Female rats have more “sweet-best” gustatory neurons than males [44], which might explain the preferences of the females in the pre-test assuming that these sex-dependent differences also exist in mice. As the male mice in the present study had no choice of selecting a diet, all dietary regimes were accepted similarly with a small preference for the initial gluten-ATI-free diet, comparable to the pre-test experiment. Nevertheless, body weight development was similar in all groups and thereby, side-effects due to food avoidance and thus developmental impairment at adolescence were not observed.

Nutritional compounds might also affect circadian system organization in rodents as exemplified by a high fat diet that blunts feeding/fasting cycles [45,46]. Gluten with or without different amounts of ATIs, had no significant effect on the distribution and amount of activity phases in the 5xFAD mice. A previous study reported increased energy expenditure in Tg2576 mice as measured by oxygen consumption at three months of age when compared to wild type mice [47]. Our study reveals that such a metabolic dysfunction evoked by genotype (which we also observed for 5xFAD, data not shown) can be further accelerated by diet: in 5xFAD mice on a gluten-containing diet, the higher dose of ATIs led to a profound increase of oxygen consumption throughout the dark and the light phase, indicating an worsened metabolic state. Whether this also goes along with reduced serum leptin levels (as found for the Tg2576 mice [47]) has not been analyzed in the 5xFAD mice. However, as they did not display reduced body weight at the here investigated age, it is not highly plausible.

Oxygen consumption, which can detect metabolic dysfunction, is also modulated by the gastrointestinal tract. Thus, the ventilatory response to hypercapnia was suppressed by antibiotic-induced disruption of the intestinal microbiota [48]. While a low concentration of ATIs in a gluten-free diet led to a small increase in villus length, villous atrophy in both the duodenum and terminal ileum was observed when mice were fed high ATI concentrations combined with gluten as compared to the group exposed to low ATIs. The finding of mild villous atrophy in the small intestine is a hallmark of celiac disease that can be cured or alleviated by a gluten-deficient diet (e.g., [49]). Here, ATIs in the presence of gluten seem to promote villous atrophy, as shown in human celiac biospies and in a celiac mouse model [12,50], even if the investigated 5xFAD mice do not have the necessary genetic background for celiac disease. In a previous investigation, a single oral challenge with ATIs did not result in morphological changes up to 24 h later, while the expression of intestinal cytokines and chemokines increased along with elevated numbers of resident macrophages in the terminal ileum [13].

Thus, the observed modest increase in intestinal myeloid immune cell subsets and the increase of CCL2 in the group of mice ingesting gluten and high ATIs versus low ATIs in our present study is in line withour former study [13]. In the present study, IL−1β expression was not increased. This is likely due to the late time point of measurement after 8 weeks of diet-induced inflammation, since the CCL2 gene is a down-stream target of IL1β [51] which is only upregulated up to 4 h after ATI exposure [13].

ATIs have been shown to affect the intestinal immune system in two major ways: (1) by activating TLR4 in intestinal lamina propria myeloid cells [12,13,25], and (2) by directly changing the intestinal microbial communities towards pro-inflammatory dysbiosis [26].

An ATI-containing diet, for example, promoted expansion of microbial taxa that are associated with the exacerbation of intestinal inflammation in dextran sodium sulfate-induced colitis in C57Bl6 mice, and this effect was due to a direct antimicrobial action of ATIs on beneficial microbiota [26]. In our dietary experiment, the strongest effect on, e.g., the Bacteroidetes/Firmicutes ratio, was found when comparing mice on a diet without gluten and ATIs as compared to those on a gluten-free diet with a low amount of ATI. No further statistically significant effect could be observed when adding gluten or increasing ATI to the diet. However, the reduced occurrence of Bifidobacteria is exactly what has also been found in the feces of celiac disease patients [52,53] and in a mouse model of inflammatory bowel disease [26]. In contrast, exogenously added Lactobacilli strains were able to counterbalance the ATI-induced inflammation and intestinal dysfunction in celiac mice [50]. However, the quantity of commensal Lactobacilli was not elevated, indicating that the metabolites produced by the added Lactobacilli were beneficial, whereas the endogenous microbial ecosystem in the here presented study was already too disturbed to counteract the pro-inflammatory action of ATIs or to promote degradation and inactivation of the ingested ATIs.

The first data indicate that the inflammatory signals evoked by ATIs are then delivered to extraintestinal organs either via ATI-activated myeloid cells that migrate out of the intestine to distant sites, and/or intestinal (microbial) metabolites that reach these distant organs. Nevertheless, a more indirect impact via changes in immunological status seems also plausible as systemic inflammation is believed to promote hallmarks of the disease (reviewed in [54]), and has also been demonstrated in a recent human study of patients with familial Mediterranean fever, a genetic inflammatory predisposition, randomized to an ATI/wheat-free vs ATI/wheat-containing diet [55].

To prove the hypothesis that the peripheral effects of ATIs might exert deleterious outcomes in diseases of the central nervous system, we investigated behavioral tasks typically applied to monitor pathogenesis of AD in mice. Indeed, the supplementation of the gluten-containing diet with the high amounts of ATIs resulted in impaired behavioral performance: nest building ability and cue-dependent fear conditioning were negatively affected. Nest building depends on the integrity of the hippocampus while especially the cue-dependent fear conditioning has been described also to be amygdala-dependent [56,57]. The degenerative processes within the hippocampus have extensively been described for AD (for a meta-analysis on hippocampal atrophy see: [58]); however, the amygdala seems to present a locus of synergistic misfolding that needs more attention [59]. Concentrations of Aβ40 and Aβ42 in the amygdala were even higher than those in the hippocampus of APP/PS1 mice and BDNF/TrkB signaling molecules were already reduced at 4 months of age in 5xFAD mice [60]. Therefore, effects on cued-fear conditioning might occur due to the involvement of amygdala at the age of the mice investigated here, while context-dependent conditioning is not yet altered. This hypothesis is also in accordance with the mild and nonsignificant increase of Aβ-positive deposits in hippocampal brain regions that were found in mice on a gluten-containing/high ATI diet. In the cortex, however, an increase of Aβ positive deposits already reached statistical significance. A contribution of an altered immune status within the brain parenchyma is not (yet) obvious from the here presented data. Expression of IL1β and CCL2 is increased in AD [61,62], and both have been discussed as pathogenicity factors and biomarkers for, e.g., progression from mild cognitive impairment (MCI) to AD [63,64,65]. Despite the increased CCL2 in the small intestine due to ingestion of ATIs, no increase of either immunological marker at the mRNA level was found in whole brain preparations. However, the whole brain preparation may have diluted an increase in signal, since in AD single local areas of the brain, such as hippocampus or amygdala are primarily affected in early stages. Therefore, a more brain-region selective PCR and prolonged dietary treatment may better reveal AD-typical inflammatory changes.

In summary, we here provide first evidence that a diet containing ATIs comparable to average human daily wheat consumption is able to aggravate pathological hallmarks and dysfunction in an AD mouse model. A final conclusion, what specific signal transduction finally evoked the aggravated behavioral impairments cannot be drawn from this study as many factors might have been involved and have been shown to be affected: immune reaction within the gut, gut architecture and thereby probably also functionality, and microbiota composition. Unraveling which molecular pathways are key to transfer the deleterious effects of ATIs from the gut towards the brain have to be investigated in future projects. In addition, AD mouse models only poorly reflect the complex human disease; nevertheless, our findings provoke the idea that neurodegenerative processes in people at risk of AD could be attenuated by reducing the amount of ATI/wheat intake. Previous data indicate that essentially all crops and foods that are gluten-free, including amaranth, rice, oats or corn, have no or a very low ATI bioactivity [13], and thereby could serve as basis for an AD nutritional therapy.

## 4. Materials and Methods

### 4.1. Experimental Animals

5XFAD [18] mice (B6SJL-Tg(APPSwFlLon,PSEN1*M146L*L286V)6799Vas/Mmjax, Jackson Lab, Bar Harbor, ME, USA) were maintained on a C57BL6/J background by cross-breeding male heterozygous animals to wild type females. For experiments, male transgenic animals aged 4 weeks were used (in total: 39 mice with 8–10 per diet; animals used for specific investigations are indicated within the respective Figure legend). Mice were firstly subjected to 4 weeks of feeding on a customized gluten- and ATI-free diet (Ssniff, Soest, Germany; see Table S1 for detailed description of composition) with a defined carbohydrate and protein (casein) content as described before [12] to achieve a basic start condition. Afterwards, animals were randomly assigned into four groups: (1) remaining on a gluten and ATI-free diet (designated Glu-/ATI-); or receiving (2) the gluten-free diet with low ATI (Glu-/ATI low (0.2% of chow, 1% of protein)); (3) diet with 25% of protein consisting of gluten, de-enriched from ATI (Glu+/ATI low (0.04% of chow, see [66])); (4) the gluten-diet, containing ATI (Glu+/ATI high (0.6% of chow)). Animals were kept on the respective diet for 8 weeks; before sacrifice at the age of 16 weeks, metabolic parameters were assessed and behavior tested (Figure 1a). All experimental procedures were carried out according to the European Communities Council Directive regarding the care and use of animals for experimental procedures and approved by local authorities (State of Rhineland-Palatinate; G14–1–087, approval date: 17 February 2015). Sample sizes were estimated by former experiences with dietary experiments (e.g., [67]).

### 4.2. Measurement of Circadian Rhythm and Metabolism (Metabolic Cages)

After feeding for 4–5 weeks on the distinct diets, mice were subjected for 48 h in metabolic cages (TSE Systems, Bad Homburg, Germany) to measure oxygen consumption as well as activity. For calculations, the inner span of 24 h was used to exclude times of high activity due to initial adaptation. During the time in the metabolic cages, mice received the same kind of diet as before.

### 4.3. Behavior Analysis

#### 4.3.1. Nest Building

For the assessment of nest building capability, animals were single caged and habituated to the nesting material used for scoring (Sizzle Pet Nesting Material, Claus GmbH, Limburgerhof, Germany; 10 g per cage) for 24 h in the presence of their former nest building material (paper towel). Animals then received fresh nesting material for three days. Subsequently, animals were deprived for 24 h to enhance nest building motivation. On day six, cages were supplemented with fresh nesting material to allow nest building for overnight. The following morning, the quality of the nest was scored as described in [30] (e.g., 0: no nest built; 5: perfectly closed dome built). Additionally, the nesting material that has not been included in the nest was weighed.

#### 4.3.2. Fear Conditioning

Fear memory was assessed in a standard auditory fear conditioning task similarly to [68]. Mice were subjected to three sessions: (1) A single conditioning session (180 s) followed by tone presentation (one tone, 30 s, 9 kHz, 72 dB SPL, pulsed 5 Hz) ending with an electric foot shock (0.8 mA, 2 s, pulsed 25 Hz); (2) assessment of conditioned fear by freezing behavior 24 h later in a context-dependent retention test (180 s, same box and light conditions 125 lx), and (3) another 2 h later in a tone-dependent retention test (180 s, 9 kHz, 72 dB SPL, pulsed 5 Hz) with different light conditions (500 lx) and box presenting a new context. Activity was measured by lightbeam breaks (Multiconditioningsystem, TSE, Bad Homburg Germany).

### 4.4. Sacrifice and Tissue Collection

After 4 weeks on the basal diet, followed by 8 weeks on either of the 4 diets, animals were anesthesized with isoflurane (Forene, AbbVieDeutschland GmbH&Co.KG, Wiesbaden, Germany) and sacrificed by decapitation. All tissue specimen were quickly dissected, flushed with ice cold PBS and subjected to dry ice freezing or submerged in fixative (4% PFA). Samples without fixation were stored at −80 °C up to further usage. Fecal samples were collected from the terminal rectum and shock-frozen in liquid nitrogen.

### 4.5. IHC and Quantitation of Aβ-Dependent Staining

After drop-fixation, brain hemispheres were sagitally cut and stained with antibody 6E10 (Covance, Princeton, NJ, USA) as described before [69]. In brief, hemispheres were embedded in paraffin and cut in 2 µm thick tissue sections. Sections were stained following standard protocols using the primary antibody 6E10 (diluted 1:500 in Antigen Retrieval Buffer 1; Medac, Wedel, Germany) and DAB as the chromogenic substrate. Deposits of Aβ-containing proteins were analyzed by an experimenter blinded to the diet-assignment within three brain regions as described previously [69]. Pictures were taken by a light microscope equipped with a digital camera (EVOS XL, Life Technologies, Carlsbad, CA, USA).

### 4.6. HE Staining and Micromorphometric Analysis

HE staining was performed following routine protocols on 6 µm thick sections [12]. Two sets of slices per animal were photographed using the EVOS XL (Life Technologies) and analyzed using ImageJ (1.5.1f) with the segmented line tool. Per slice, two villus lengths and two muscularis thickness values were assessed. Measures were taken as pixels and are indicated as arbitrary units (mean ± SEM).

### 4.7. Microbiome Analysis

Fecal samples were analyzed by the Institute of Microecology (Herborn, Germany) as described before [70]. In brief, microbial DNA was extracted from feces using the QIAsymphony DSP Virus/Pathogen Mini-Kit on the QIAsymphony SP (QIAGEN, Hilden, Germany). Primers were selected to recognize either the whole bacterial phyla (Firmicutes, Bacteroidetes) or in the case of Bifidobacteria, Lactobacilli, and Enterococci were the main representatives within the murine microbiome. PCR amplification and detection were performed using a Rotor-Gene Q system (Applied Biosystems, QIAGEN, Hilden, Germany).

### 4.8. RNA Preparation and qPCR

Both procedures were performed as described previously [12]: RNA was extracted from the terminal ileum (E3598–02; Roboklon, Berlin, Germany) and reverse-transcribed (High Capacity cDNA Reverse Transcription Kit, Thermo Fisher Scientific, Waltham, MA, USA). Quantitative polymerase chain reaction (qPCR) was performed using exon–exon boundary-spanning primer sequences (Table S3) and the SYBR Green methodology on a Step One Plus sequence amplification system (Applied Biosystems, Foster City, CA, USA). The relative mRNA expression of the tested gene normalized to beta actin expression was calculated using the ΔΔCt method.

### 4.9. Statistical Analysis

For comparison of two groups, data were analyzed by multiple t-test. One-way analysis of variance (ANOVA) was performed for comparison of the 4 diet groups, followed by the indicated post hoc test. *p*-values < 0.05 were considered statistically significant and results were presented as mean ± SEM. Data analyses were performed using GraphPad Prism 6 and 8 (Graph Pad Software, La Jolla, CA, USA). Normality test was performed by Anderson–Darling and Shapiro–Wilk test. In case of morphometric analysis, data did not pass these tests; therefore Kruskal–Wallis test with Dunn’s multiple comparison test was performed.

## Supplementary Materials

Supplementary materials can be found at https://www.mdpi.com/1422-0067/21/17/6288/s1.

## Abbreviations

Aβ AD ATIs	Amyloid beta peptide Alzheimer’s disease Amylase trypsin inhibitors
CCL2	C–C motif chemokine ligand 2
5xFAD	Transgenic AD mouse model with 5 familial mutations
IL1β	Interleukin 1 beta
TLR	Toll-like receptor
