# Supplementary material for: Dietary Wheat Amylase Trypsin Inhibitors Impact Alzheimer’s Disease Pathology in 5xFAD Model Mice
Source: Int J Mol Sci. 2020 Aug 31;21(17):6288. doi: 10.3390/ijms21176288 (PMC7503408; doi:10.3390/ijms21176288)
Supplement: Supplementary file 1 [file ijms-21-06288-s001.pdf]

Suppl. Table 1. Dietary composition of customized gluten- and ATI-free diet (percentage of ingredients and amino acids).

Gluten- and ATI-free diet (S8251-E030)			
**Ingredients**	**Percentage (%)**	**Amino acids**	**Percentage (%)**
Wheat Gluten (Sigma (>80%CP)	-	Lysine	1.7
Casein	20.3	Methionine	1.0
Cornstarch. pre-gelatinized	-	Met & Cys	1.1
Maltodextrin	-	Threonine	1.0
Corn. pre- treated	21	Tryptophan	0.3
Rice meal	41	Arginine	1.0
Sucrose	2	Histidine	0.7
Lignocellulose	2	Valine	1.5
pur. Cellulose powder	3	Isoleucine	1.2
L-cystine	-	Leucine	2.3
L-Lysine HCI	-	Phenylalanine	1.2
DL-Methionine	0.2	Phe & Tyr	2.2
Mineral & Trace element premix	6	Glycine	0.6
Vitamin premix	1	Glutamic acid (+Gln)	4.9
Choline chloride	0.3	Aspartic acid (+Asn)	1.8
Soybean oil	3.3	Proline	2.3
		Serine	1.3
		Alanine	0.9

Suppl. Table 2. Statistical comparison of oxygen consumption over the 24h measurement period. P-values <0.05 derived by multiple t-test are indicated by a *.

gluten-free without ATI versus low ATI			gluten-containing low versus high ATI			gluten-free without ATI versus low ATI			gluten-containing low versus high ATI		
time of day		*p*-value	time of day		*p*-value	time of day		*p*-value	time of day		*p*-value
18:00	*	0,040	18:00	*	0,006	06:00		0,528	06:00		0,197
		0,289			0,226			0,552		*	0,009
	*	0,044		*	0,016		*	0,034		*	0,004
		0,218			0,095			0,191		*	0,005
19:00		0,914	19:00	*	0,013	07:00		0,094	07:00	*	0,007
		0,114		*	0,021			0,431			0,086
	*	0,036		*	0,008			0,609			0,144
	*	0,026		*	0,004			0,735		*	0,012
20:00		0,071	20:00	*	0,009	08:00		0,752	08:00	*	0,005
		0,080		*	0,006			0,408			0,101
		0,130		*	0,004			0,873		*	0,015
		0,326		*	0,045			0,380		*	0,015

21:00	*	0,024	21:00	*	0,008	09:00		0,176	09:00	*	0,018
		0,065		*	0,002			0,131		*	0,015
	*	0,028		*	0,011			0,328		*	0,044
		0,109		*	0,036			0,533			0,743
22:00		0,051	22:00	*	0,042	10:00		0,961	10:00		0,440
	*	0,016		*	0,006			0,256			0,101
		0,055		*	0,014			0,294			0,094
	*	0,008		*	0,001			0,430			0,052
23:00		0,055	23:00	*	0,002	11:00		0,633	11:00		0,058
		0,089		*	0,001			0,756		*	0,020
		0,333		*	0,017			0,427		*	0,032
		0,186		*	0,030			0,426			0,087
00:00		0,057	00:00	*	0,003	12:00		0,648	12:00	*	0,039
		0,082		*	0,001			0,439			0,182
		0,213		*	0,003			0,227		*	0,006
		0,167		*	0,004			0,362		*	0,031
01:00		0,467	01:00	*	0,010	13:00		0,517	13:00	*	0,005
		0,340			0,202			0,675		*	0,007
		0,154			0,123			0,730		*	0,031
		0,360		*	0,014			0,172			0,074
02:00		0,970	02:00	*	0,017	14:00		0,529	14:00		0,077
		0,439		*	0,001			0,907			0,213
		0,677		*	0,006			0,711			0,575
		0,174		*	0,000			0,374			0,289
03:00	*	0,029	03:00	*	0,000	15:00		0,628	15:00		0,119
		0,084		*	0,008			0,242		*	0,016
		0,439			0,051			0,208			0,052
		0,761			0,188			0,215			0,372
04:00		0,798	04:00		0,101	16:00		0,277	16:00		0,165
		0,997		*	0,024			0,502			0,154
		0,768		*	0,040			0,739		*	0,041
		0,806		*	0,016			0,464		*	0,031
05:00		0,489	05:00	*	0,001	17:00		0,105	17:00	*	0,002
		0,819		*	0,024		*	0,015		*	0,000
		0,816		*	0,039			0,598			0,060
.		0,490			0,095			0,987			0,195

Suppl. Table 3. SYBR Green primer sequences (Eurofins, Hamburg, Germany).

Genes	Primers (F, Forward and R, Reverse)
IL1β	F caa cca aca agt gat att ctc cat g
	R gat cca cac tct cca gct gca
CCL2	F ccc aat gag tag gct gga ga
	R tct gga ccc att cct tct tg
beta actin	F ggc att gtt acc aac tgg gac gac
	R cca gag gca tac agg gac agc aca g

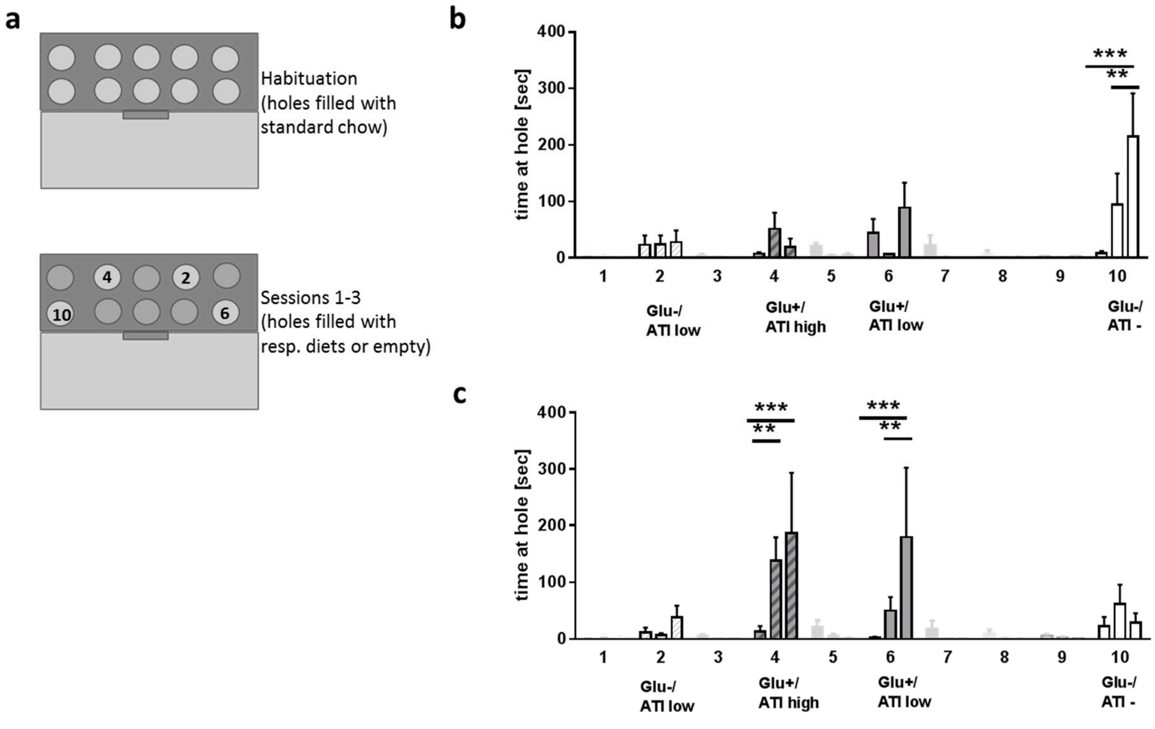

Suppl. Figure 1. Food preference in male and female 5xFAD mice. (**a**) Mice were habituated to a two-chamber containing an entry box (light grey) and a hole-board (dark grey) with each hole filled with standard diet on day one. Mice were introduced in the entry chamber and the door was opened after 1 min to allow access to the hole-board for 10 min. The following three days, mice (**b**: male; **c**: female; n=4 each) were presented the four different diets in the indicated holes (2,4,6,10) and time spent at each hole within the 10 min was recorded with an automated camera system (EthoVision 8.5, Noldus software, The Netherlands)). The position of each diet was kept during the whole test to allow memorizing palatable food. Data were analyzed by Two Way ANOVA with Tukey's multiple comparisons test (**, p<0.01; ***, p<0.001) and are presented as mean of time + SEM for each hole at each of the testing days.
